# Relationship between C-reactive protein/albumin ratio and mucocutaneous symptom frequency and disease severity in Behçet’s disease

**DOI:** 10.55730/1300-0144.5803

**Published:** 2024-02-12

**Authors:** Erdal PALA, Mustafa BAYRAKTAR

**Affiliations:** 1 Department of Skin and Venereal Diseases, Faculty of Medicine, Atatürk University, Erzurum, Turkiye; 2 Department of Family Medicine, Faculty of Medicine, Atatürk University, Erzurum, Turkiye

**Keywords:** Behçet’s disease, serum albumin, C-reactive protein, C-reactive protein/albumin ratio, severity of illness index, inflammation

## Abstract

**Background/aim:**

There is no specific marker that can be applied to determine the severity of Behçet’s disease. The aim of this study is to investigate the potential of C-reactive protein (CRP)/albumin (CAR) ratio as a tool for assessing the severity of Behçet’s disease.

**Materials and methods:**

A retrospective crosssectional study was conducted by examining hospital archives. The CRP and albumin levels of Behçet’s disease patients who presented to our dermatology clinic over a three-year period from February 2020 to February 2022 were included, along with the identical laboratory parameters in the control group. The CAR ratio was calculated and statistically compared across different clinical features of the disease and with the control group.

**Results:**

Of the 97 patients with Behçet’s disease, 70.1% (n = 68) were female and the median age was 36.0 years (IQR = 20.5), whereas of the 53 control subjects, 77.4% (n = 41) were female and the median age was 35.0 years (IQR = 19.0). There was no statistically significant difference in sex or age between the groups (p > 0.05). The levels of CRP and CAR were found to be significantly elevated in patients with Behçet’s disease compared to the control group (both p < 0.001). According to the ROC analysis, the area under the curve (AUC) of CRP level and CAR were found to be 0.784 (95%CI: 0.710–0.859), and 0.786 (95%CI: 0.712–0.861), respectively. The cut-off value for CRP was determined as 1.485, whereas for CAR it was 0.324. As the severity of Behçet’s disease increased, there was a statistically significant increase in the CAR level (p < 0.001). The severity of Behçet’s disease was statistically significantly associated with a high CAR level.

**Conclusion:**

CAR can be used as a quick and easily calculated parameter to assess the severity of Behçet’s disease.

## 1. Introduction

Behçet’s disease (BD) is an autoimmune, autoinflammatory, lymphocytic infiltration of vessels of all sizes, characterized by remissions and exacerbations, primarily affecting endothelial cells. The classic triad of the disease, which can progress with different clinical findings, includes oral aphthous ulcer, genital ulcer, and uveitis. While the disease may progresses with only mucocutaneous findings, it can also have severe systemic involvement [1]. The etiopathogenesis of BD has not been fully explained. Although many mechanisms such as genetic, epigenetic, infectious, immunological, and environmental factors have been emphasized, it is generally accepted that BD is an autoinflammatory disease in terms of its pathogenesis and general characteristics and [2].

C-reactive protein (CRP) is classified as an acute positive phase reactant with very high sensitivity in detecting inflammation and tissue damage. CRP levels are elevated in infections, inflammatory diseases, trauma, malignancy, and cardiac pathology [3]. On the other hand, albumin is a negative acute-phase reactant. Its levels may decrease in various inflammatory diseases, liver, and kidney diseases, or in cases of malnutrition. [4]. In recent years, the CRP/albumin ratio (CAR) has gained popularity as a parameter indicating the inflammatory state. It is obtained by calculating the ratio of acute positive phase protein CRP ratio to the acute negative phase protein albumin. Although CRP and albumin are used alone to evaluate the severity and activation of some diseases, it has been found that the use of CAR as a biomarker is more reliable than their use alone. This is because CRP and albumin plasma levels can be affected by many factors [5].

There are some studies in the literature on inflammatory markers that can be used in BD. CRP, erythrocyte sedimentation rate, neutrophil to lymphocyte ratio, and platelet to lymphocyte ratio have been studied in Behçet’s disease to indicate disease activity [6, 7]. Although these parameters are helpful in reflecting the activation of the disease, they are not specific to BD and, as BD progresses with exacerbations and remissions, they are not sufficient to reflect the severity of the disease. Therefore, there is a need for an inflammatory parameter to determine the severity of BD, and the applicability of CAR, which is a practical and easily applicable parameter, should be investigated. The aim of this study was to investigate the association of CAR with the severity of disease and clinical findings in BD.

## 2. Materials and methods

### 2.1. Study design

This research was conducted as a cross sectional, retrospective, single-center study.

### 2.2. Setting

The research was conducted between February 2020 and February 2022 in a dermatology clinic at a tertiary university hospital serving approximately 4.5 million people in eastern Türkiye, and control group data were obtained from a family health center.

### 2.3. Participants

The study included 97 patients with inactive or active Behçet’s disease, aged 18–75, diagnosed in accordance with the diagnostic criteria of International Behçet’s Disease, who applied to the dermatology Behçet outpatient clinic of our hospital between February 2020 and February 2022. Patients without any symptoms were considered inactive, in remission. As a control group, 53 healthy individuals with similar demographic characteristics from a family health center were enrolled. Demographic data of the patient and control groups were recorded. Patients younger than 18 years, with acute infections, nutritional problems, malignancy, symptomatic gastrointestinal disorders, renal and hepatic diseases were excluded. As the study was retrospective and designed as an archive review of the files of all patients, it was neither necessary nor possible to acquire informed consent from the patients.

### 2.4. Experimental

The archive files of the patients were retrospectively reviewed, and their symptoms as of the date of admission, along with CRP and albumin values, were obtained in accordance with this date. CAR was calculated and recorded in both the patient and control groups. In evaluating the laboratory analyses conducted, the laboratory values of our hospital were used as reference values for the test results. In this context, the following values were considered normal: CRP <5 mg/L, albumin 3.5–5.2 g/dL. The biochemical analysis of CRP and albumin was performed using the electrophotometric method in a Roche instrument.

### 2.5. Disease severity of Behçet’s disease

The severity of BD was determined using Krause’s BD clinical severity scoring. Accordingly, one point was assigned for each mild symptom (such as oral aphthous ulcer, genital ulcer, arthralgia, erythema nodosum, folliculitis, papulopustular lesions), two points were allocated for each moderate symptom (including arthritis, deep vein thrombosis in the legs, gastrointestinal involvement, anterior uveitis), and patients’ scores were obtained by assigning three points for each severe symptom (such as retinal vasculitis, posterior uveitis/pan uveitis, neuro-Behçet’s, bowel perforation, arterial thrombosis). Patients were classified into three distinct groups based on the total score obtained by summing the scores. The severity of the disease was determined as follows: severe (≥7), moderate (4–6), and mild (<4).

### 2.6. Statistical analysis

The data were analyzed using IBM SPSS Statistics 23.0 software (IBM Corporation, Armonk, NY, USA). Categorical data were presented as percentages and frequencies, while numerical data were presented as means and standard deviations. In addition, the chi-square test was preferred in the analysis of categorical data. The Shapiro-Wilk test was employed to assess the adherence of the numerical data to a normal distribution. In the analysis of two independent groups, the Student’s t-test was employed when the data followed a normal distribution. Conversely, the Mann-Whitney U test was utilized when the data did not exhibit a normal distribution. In the analysis of three or more groups, one-way analysis of variance (ANOVA) test was used if normal distribution was provided, and Kruskal-Wallis test was used if normal distribution was not provided. The Tukey test was used for posthoc analysis when a significant difference was found in the comparison of three or more groups. Spearman’s correlation analysis was carried out in the correlation analysis of the two numerical groups. Receiver operating characteristic (ROC) analysis was used to calculate the area under the curve (AUC) and to determine the cut-off value. p < 0.05 was considered statistically significant throughout the study.

## 3. Results

Of the 97 Behçet’s disease patients and the 53 healthy control group included in our study, 70.1% (n = 68), and 77.4% (n = 41) were female, respectively, and there was no significant change (p > 0.05). While the median age of the Behçet group was 36.0 years with an interquartile range (IQR) of 20.5 years, the median age of the control group was 35.0 years (IQR = 19.0) and no significant change was found (p > 0.05) (Table 1).

**Figure 1:**
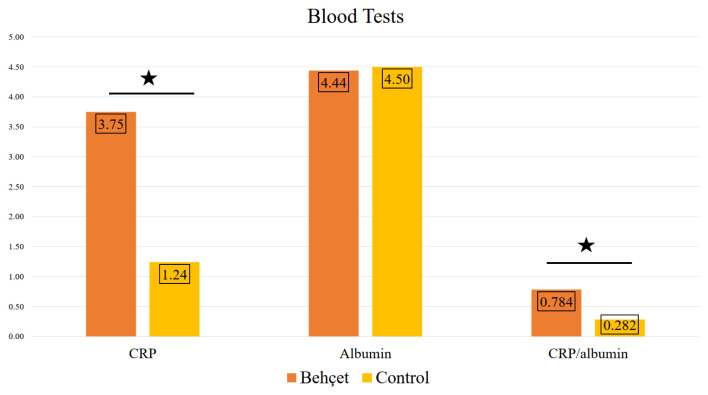
Comparisons of median values of CRP, albumin, and CRP/albumin ratio of Behçet’s disease and control group. (Note: Asterisk indicates statistical difference between two groups at p < 0.001.)

The levels of CRP and CAR in the Behçet group were significantly higher than those in the control group (p < 0.001) (Table 1) (Figure 1). The clinical findings of the participants in the Behçet group and their relationship with CRP and CAR are presented in Table 2. When the disease activity of Behçet’s disease patients was classified as active or inactive, CRP, albumin, and CAR were not statistically different (p = 0.081, p = 0.771, p = 0.062, respectively). When the severity of the disease in Behçet’s disease patients was classified according to Krause’s clinical severity scoring, it was found that as the disease severity increased, the levels of CRP and CAR increased statistically (both p < 0.001), but the albumin level did not cause a significant difference (p = 0.911). In the correlation analysis, a strong positive correlation occurred between the CAR and the severity of the disease (r = 0.478, p < 0.001).

**Figure 2:**
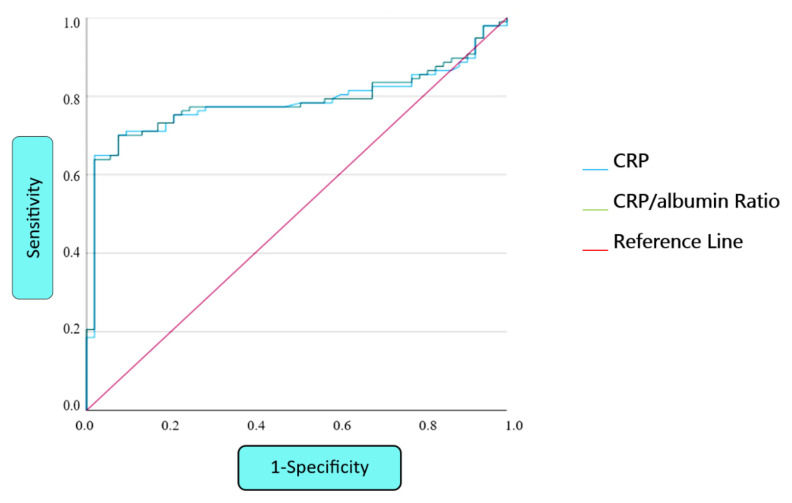
ROC curves of CRP and CRP/albumin ratio in Behçet’s disease.

When statistical comparisons were made between the findings of BD and albumin, CRP and CAR levels, the presence of papulopustular lesion, the presence of uveitis and uveitis activity, and the elevation of CRP and CAR levels were statistically significantly higher (p < 0.05). According to joint involvement, the presence of arthritis was significantly associated with high CRP, low albumin, and high CAR (p < 0.05) (Table 2). There is a remarkable statistical association between the number of skin and mucosal involvement and high CRP and CAR. After correlation analysis, it was found that CRP and CAR were positively correlated with the number of involvements (r = 0.293, p = 0.004; r = 0.303, p = 0.003, respectively). The ROC curves obtained from the ROC analysis of CRP and CAR are presented in Figure 2. Accordingly, the area under the curve (AUC) of the CRP level was found to be 0.784 (95%CI: 0.710–0.859). The AUC value for CAR was found to be 0.786 (95%CI: 0.712–0.861) (Table 3). The cut-off for CRP was 1.485 (with 75.3% sensitivity and 73.6% specificity), while the cut-off value for CAR was 0.324 (with 76.3% sensitivity and 75.5% specificity).

## 4. Discussion

BD is a chronic relapsing multisystemic disease whose etiopathogenesis has not been fully elucidated, with mucocutaneous findings, as well as systemic involvement with high morbidity and mortality such as eye, neurological system, gastrointestinal system, joint, and vascular involvement [1].

This study was the first in the literature to investigate the usability of CAR as a promising, simple, and practical inflammatory biomarker in BD. According to the data we obtained, there was a statistically significant increase in CAR and the severity of BD and clinical findings, and that it could be used in the detection of the disease with high sensitivity and specificity. Since BD is accepted as an autoinflammatory disease, there is still no specific parameter to monitor disease activity and severity. A review of the literature shows that only the relationship between the presence of uveitis and CAR in BD has been investigated [8], and no study examining the relationship between disease severity and other clinical findings could be found. Therefore, parameters with high specificity and sensitivity that can be easily and quickly applied are needed to monitor the severity of this disease, which has a high morbidity and mortality. CRP is a well-known positive acute phase reactant with a short half-life. It begins to increase in the early stages of inflammation. It is synthesized in hepatocytes and given to the blood by the effect of cytokines such as tumor necrotic factor-alpha (TNF-α), interleukin-1 (IL-1), and especially IL-6. In the case of acute inflammation, it activates the classical complement pathway, but also initiates cellular immunity by binding to the Fc part of IgG. While CRP increases the synthesis of adhesion molecules, it decreases nitric oxide (NO) release in endothelial cells. Simultaneously, it helps the adhesion of leukocytes to the endothelium by providing protein-1 activation from monocyte chemoattractant. Thus, it contributes to the formation of vascular pathologies [9]. Although there are studies showing that CRP is useful in distinguishing between inactive and active BD, CRP levels may vary among BD patients with different symptoms. While CRP was found to be high in erythema nodosum and vascular symptoms in one study, it was found to be high especially in vascular involvement in another study [6, 10]. Since different CRP levels are observed in different clinical findings of BD, the role of this parameter in reflecting the severity of the disease remains controversial. Our study found that CRP levels were found to be significantly higher in patients with papulopustular lesions, joint involvement, and uveitis symptoms. However, in our study, 86.6% (n = 84) of the active Behçet’s disease patients and 13.4% (n = 13) of the inactive ones were found to be inactive. The difference in CRP levels between active and inactive patients was not statistically significant (p > 0.05). However, when evaluating the disease in terms of severity, a statistically significant difference was found between the CRP levels of severe BD and mildly severe BD (p < 0.001). The area under the curve (AUC) for CRP levels obtained through ROC analysis was found to be 0.784 (95%CI: 0.710–0.859). Albumin is a protein that is most abundant in plasma and functions as an antioxidant by reducing the production of reactive oxygen radicals. It also acts as a carrier for many substances [11]. During inflammation, albumin synthesis decreases, while its degradation increases. Again, there is a decrease in the amount due to transcapillary escape [12]. BD is also a chronic inflammatory disease, and albumin levels may decrease due to increased catabolism. In our study, there was no statistically significant difference in plasma albumin levels between the BD group and the control group (p > 0.05). Similarly, no significant difference occurred in a study examining the presence of uveitis and albumin levels in Behçet’s disease patients [8]. In our study, a significant decrease was found in albumin levels in BD patients who only had joint complaints compared to the albumin level of Behçet’s disease patients (p < 0.05). This decrease was attributed to a higher level of inflammation in the joint involvement. However, albumin levels cannot provide sufficient parameters for determining disease severity and activity due to the influence of various factors.

CAR is a parameter that provides information on both inflammatory status and nutritional status. It is obtained by calculating the ratio of the acute positive phase protein CRP to the acute negative phase protein albumin. This parameter has recently been used in dermatological diseases. According to a study [13], CAR was found to be higher in psoriasis vulgaris patients compared to the control group. Furthermore, there have been studies on CAR in numerous diseases beyond dermatology. CAR has been used to evaluate diagnosis and treatment, predict prognosis and mortality in various patient groups, including those with inflammatory diseases, sepsis, intensive care patients, malignancies, and coronary artery diseases. High CAR rates have been associated with poor prognosis and increased mortality [14–17]. Various studies have investigated CAR in relation to BD [18, 19], but it has never been compared to the severity of the disease.

Our study found that the CAR was significantly higher in the BD group than in the control group (p < 0.001). The AUC value of CAR was found to be 0.786 (95%CI: 0.712–0.861), indicating that the CAR value with a cut-off value of 0.324 can be used as a useful parameter in BD.

Examining the relationship between BD clinical findings and CAR, it was found that CAR levels were statistically significantly higher in patients with papulopustular lesions, joint involvement, and uveitis (all p < 0.05). These results indicate that inflammation is significantly more intense in Behçet’s disease patients with these clinical findings, and CAR may be useful in following the clinical course of these symptoms.

In a study conducted on 50 Behçet’s disease patients, the relationship between CAR and uveitis was examined. It was found that CAR was highly correlated with the severity of inflammation in those with uveitis [8]. Similarly, in our study, CAR was statistically higher in patients with uveitis (p < 0.001). These results suggest that CAR is a useful parameter for evaluating uveitis activity in BD patients.

In a study comparing the severity of ulcerated lesions and CAR in patients with recurrent aphthous stomatitis, it was found that CAR was higher in patients with active oral ulcer [20]. However, our study did not find a statistically significant relationship between CAR and patients with oral aphthous ulcer (p = 0.984). In our study, the number of skin and mucosal symptoms was compared with the CAR level, and a positive mild to moderate correlation was found between the number of skin and mucosal symptoms (r = 0.303, p = 0.003).

Several severity indices can be used to evaluate clinical severity in BD. However, there is no standard disease severity index. One such index is Krause’s clinical severity scoring for BD [21]. In our study, we used Krause’s BD clinical severity scoring to determine the severity of the disease. Accordingly, 13.4% (n = 13) of the 97 Behçet’s disease patients were asymptomatic, and the disease severity index was not calculated for them. Among the 84 symptomatic Behçet’s disease patients for whom the disease severity index was calculated, 56.7% (n = 55) were classified as mild, 18.6% (n = 18) as moderate, and 11.3% (n = 11) as very severe. When examining the relationship between disease severity and CAR, it was found that as disease severity increased, CAR also increased significantly (p < 0.001), while the albumin level did not differ significantly (p = 0.911). A strong positive correlation was observed between CAR and disease severity (r = 0.478, p < 0.001). In clinical practice, CAR can be considered a valuable parameter for monitoring the severity of the disease, and it can be recommended as a guiding parameter for clinicians in selecting treatment based on the severity of the disease in Behçet’s disease patients.

In conclusion, the relationship between CAR and disease severity, as well as frequency of mucocutaneous symptoms, has been investigated for the first time in this study. Additionally, the study was conducted in Behçet’s disease patients with a relatively high participation rate and compared them with a control group. The level of CAR was more remarkable in patients with Behçet’s disease than in the control group, and CAR increased with increasing disease severity. Overall, CAR seems to be a useful parameter to monitor the severity of some symptoms of the disease. Based on these results, we believe that CAR may be a promising, new, sensitive, and specific inflammatory marker that can be easily and quickly applied in clinical practice in Behçet’s disease.

Our study has some limitations. First, the retrospective nature of the study is a limitation. Secondly, the fact that some patients were evaluated while receiving treatment may have affected the activation of the disease and the levels of inflammatory parameters. Thirdly, although our study was a study with a broad participation, we think that the results obtained cannot be generalized due to its single-center nature, and there is a need for prospective studies to be conducted in more than one center.

## Figures and Tables

**Table 1:** Comparison of demographic characteristics and laboratory test results of study groups.

		Behçet	Control	p
**Sex**	Women (n %)	68 (70.1%)	41 (77.4%)	0.341*
	Men (n %)	29 (29.9%)	12 (22.6%)	
	Total	97 (100.0%)	53 (100.0%)	
**Age** (year; median, IQR)		36.0 (IQR = 20.5)	35.0 (IQR = 19.0)	0.777†
**CRP** (mg/L; median, IQR)		3.76 (IQR = 7.61)	1.24 (IQR = 0.46)	**<0.001** †
**Albumin** (gr/dL; median, IQR)		4.44 (IQR = 0.42)	4.5 (IQR = 0.4)	0.097†
**CRP/albumin ratio** (median, IQR)		0.784 (IQR = 1.70)	0.282 (IQR = 0.09)	**<0.001** †

* Pearson chi-square test;

† Mann-Whitney U test.

CRP: C-reactive protein.

(Note: p values in bold indicate p is statistically significant at less than 0.05).

**Table 2:** Clinical findings of Behçet’s disease patients and their relationship with CRP, albumin, and CRP/albumin ratio.

		Frequency	Percent	p		
				CRP	Albumin	CRP/albumin Ratio
**Disease activity**	Active	84	86.6	0.081*	0.771*	0.062*
	Inactive	13	13.4			
**Disease severity**	Inactive	13	13.4	**<0.001** †	0.911†	**<0.001** †
	Mild	55	56.7			
	Moderate	18	18.6			
	Severe	11	11.3			
**Oral aphthous ulcer**	Yes	67	44.7	0.953*	0.055*	0.984*
	No	30	20.0			
**Genital ulcer**	Yes	28	18.7	0.182*	0.372*	0.155*
	No	69	46.0			
**Papulopustular lesion**	Yes	55	36.7	**0.001** *	0.793*	**0.002** *
	No	42	28.0			
**Erythema nodosum**	Yes	20	13.3	0.193*	0.082*	0.156*
	No	77	51.3			
**Superficial thrombophlebitis**	Yes	4	2.7	0.095*	0.611*	0.114*
	No	93	62.0			
**Pathergy test**	Positive	42	28.0	0.088*	0.137*	0.067*
	Negative	55	36.7			
**Number of skin and mucosa lesion**	0	10	6.7	**0.027** †	0.588†	**0.027** †
	1	21	14.0			
	2	24	16.0			
	3	28	18.7			
	4	11	7.3			
	5	3	2.0			
**Joint involvement**	None	76	50.7	**0.031** †	**0.029** †	**0.026** †
	Arthralgia	16	10.7			
	Arthritis	5	3.3			
**Uveitis**	Yes	27	18.0	**0.008** *	0.592*	**0.010** *
	No	70	46.7			
**Activity of uveitis**	Active	19	12.7	**<0.001** *	0.807*	**<0.001** *
	Inactive	8	5.3			

* Mann-Whitney U test;

† Kruskal-Wallis test.

(Note: p values in bold indicate p is statistically significant at less than 0.05).

**Table 3:** ROC analysis of CRP and CRP/albumin ratio in Behçet’s disease.

Test result variable (s)	Area	Std. error a	Asymptotic Sig. b	Asymptotic 95% confidence interval	
				Lower bound	Upper bound
CRP	0.784	0.038	0.000	0.710	0.859
CRP/albumin ratio	0.786	0.038	0.000	0.712	0.861

a Under the nonparametric assumption.

b Null hypothesis: true area = 0.5.

## Data Availability

Data and materials may be shared upon reasonable request.
